# The Crucial Function of Biomarkers in the Diagnosis and Management of Systemic Sclerosis-Associated Interstitial Lung Disease

**DOI:** 10.7759/cureus.106189

**Published:** 2026-03-31

**Authors:** Tasneem A Alqatta, Reem E Aldhaleai, Adam A Abushaala, Jordyn T Dvorak, Salim Fredericks

**Affiliations:** 1 Department of Medicine, Royal College of Surgeons in Ireland - Bahrain, Muharraq, BHR

**Keywords:** biomarkers, interstitial lung disease (ild), ssc-ild pathogenesis, systemic sclerosis-associated interstitial lung disease (ssc-ild), systemic sclerosis (ssc)

## Abstract

There is a critical need for biomarkers for systemic sclerosis-associated interstitial lung disease (SSc-ILD) due to the high mortality rate associated with interstitial lung disease in patients with systemic sclerosis. The pathology of this condition remains poorly understood, highlighting the urgency for advancements in early diagnosis and detection. Biomarkers have the potential to provide a solution to this pressing issue; however, these biomarkers must be of high quality and reliability to aid in the management of SSc-ILD. Treatments are currently initiated following the onset of frank clinical manifestations. These clinical presentations naturally occur only after significant disease progression and tissue damage. Biomarkers may allow for earlier interventions, which may prevent reduced lung function. Biomarkers facilitate diagnostic reasoning processes and prognostic predictions in personalized patient management plans. In current clinical practice, using biomarkers in SSc-ILD is far from well-established. The background and explanation of why there is a near absence of validated biomarkers in this setting are reviewed in this article. The review aims to focus on the candidate markers that are to become routinely offered by pathology services, highlighting the biochemical and pathological rationale for their use.

## Introduction and background

Systemic sclerosis (SSc) is a chronic autoimmune disease characterized by vascular dysfunction and progressive fibrosis. Interstitial lung disease (ILD) involves inflammation and scarring of the lung tissue, impairing respiratory function. When ILD develops in patients with SSc, it is referred to as systemic sclerosis-associated interstitial lung disease (SSc-ILD). SSc‑ILD is a leading cause of morbidity and mortality in SSc, with prevalence estimates ranging from approximately 35% to 90%, depending on population studies and diagnostic modalities, such as high-resolution CT (HRCT) or pulmonary function tests (PFTs) [1]. The condition often begins insidiously, manifesting as a dry cough, exertional dyspnea, bibasilar crackles, and fatigue, often preceding radiographic fibrosis on HRCT findings [2]. Early identification of at-risk individuals, for example, those with anti-topoisomerase I antibodies, diffuse skin involvement, or elevated C-reactive protein (CRP), is essential for biomarker-supported early diagnosis, prognosis, and clinical management [2]. However, despite increasing awareness and therapeutic advances, clinicians still lack reliable tools to accurately predict which patients will develop progressive lung disease and to guide timely therapeutic decision-making.

This review highlights the crucial clinical role of biomarkers in the diagnosis, monitoring, and management of SSc-ILD, focusing on well-studied markers including surfactant protein D (SP-D), surfactant protein A (SP-A), and Krebs von den Lungen-6 (KL-6) [1,2]. Clinical signs such as crackles and a decline in diffusing capacity of the lungs for carbon monoxide (DLCO) or forced vital capacity (FVC) often prompt biomarker evaluation to support early detection and disease monitoring. SSc pathophysiology involves vascular injury, immune dysregulation, and fibrosis, with ILD representing the leading cause of SSc-related mortality. Given the heterogeneity of disease progression, biomarkers are needed to improve risk stratification, support early diagnosis, and guide individualized treatment decisions.

Although SSc-ILD is a relatively uncommon autoimmune condition, it imposes a considerable clinical and economic burden worldwide. In the United States, the average annual direct healthcare cost per patient with SSc-ILD is approximately $33,000, while in Europe, countries such as Sweden and France report costs exceeding €25,000 per patient [3,4]. Most of these costs arise from hospitalizations and long-term follow-up care, particularly in patients with extensive disease. These findings highlight the urgent need for reliable biomarkers and improved monitoring strategies to enable earlier detection, guide treatment, and prevent irreversible lung injury. Therefore, this review focuses on biomarkers that can support early diagnosis, prognosis, and evidence-based clinical management strategies in SSc-ILD.

Systemic sclerosis-associated interstitial lung disease

**Figure 1:**
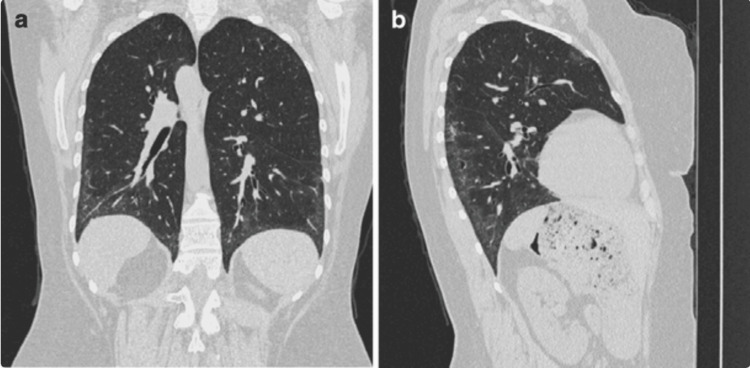
Early radiological features of SSc-ILD on chest CT. Coronal and sagittal chest CT reconstructions demonstrate the classic appearance of early lung involvement in systemic sclerosis-associated interstitial lung disease (SSc-ILD), with subtle ground-glass opacities and reticular changes predominantly in the peripheral, posterior, and basal lung regions. Reproduced from Strollo and Goldin (2010) [10]. Image reproduced from an open-access article distributed under the terms of the Creative Commons Attribution Noncommercial License.

**Figure 2:**
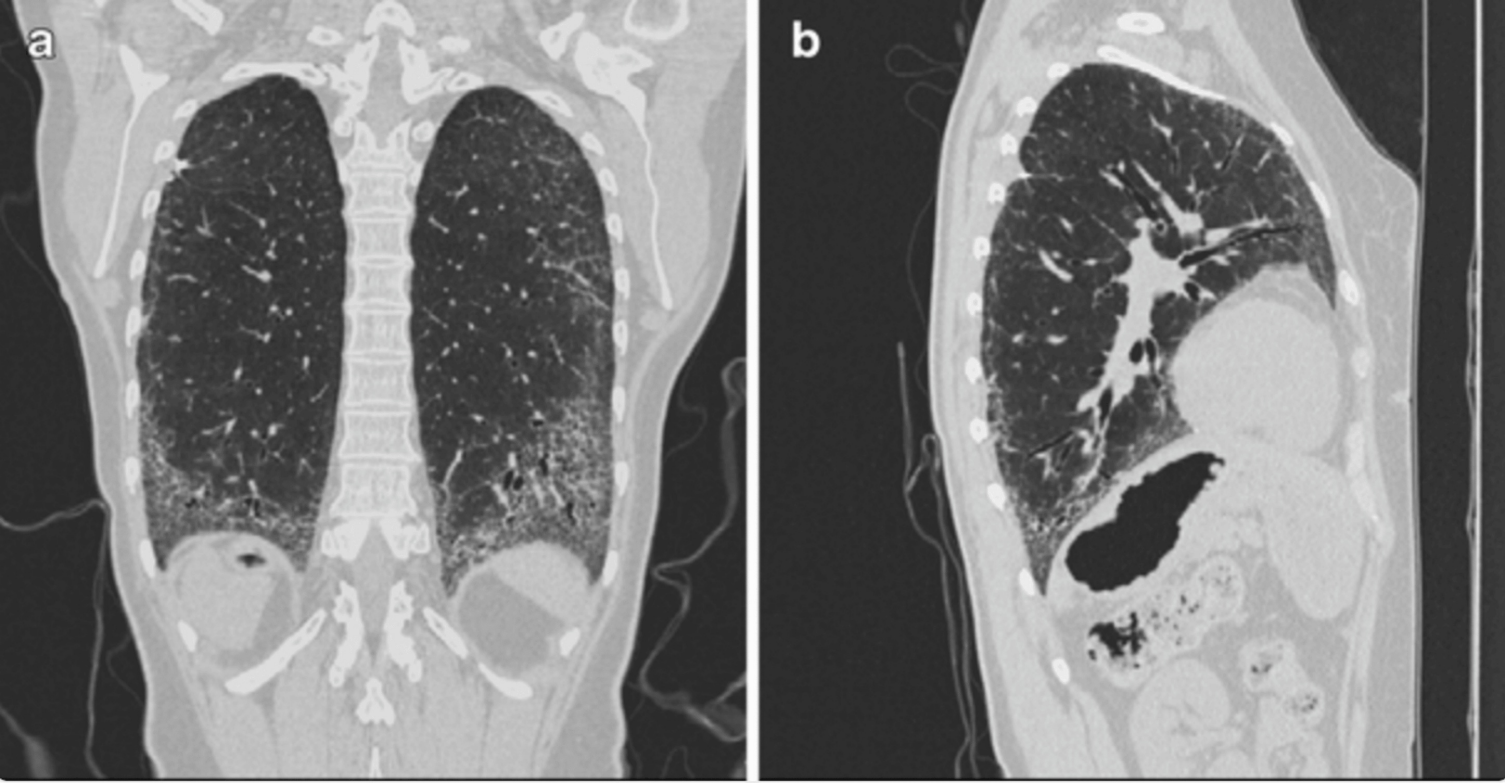
Advanced radiological features of SSc-ILD on chest CT. Coronal and sagittal chest CT reconstructions (lung window) demonstrate the classic appearance of advanced systemic sclerosis-associated interstitial lung disease (SSc-ILD) in patients with progressive disease. Findings include extensive architectural distortion due to progressive pulmonary fibrosis (a and b). Reproduced from Strollo and Goldin (2010) [10]. Image reproduced from an open-access article distributed under the terms of the Creative Commons Attribution Noncommercial License.

SSc‑ILD represents a leading cause of morbidity and mortality in SSc, affecting an estimated ≈50% of patients and accounting for up to 30% of SSc‑related deaths [5,6]. It typically arises early, often within the first five years of SSc onset, and evolves with highly variable trajectories. While some individuals experience stable disease, 20-30% progress rapidly, underscoring the need for precise risk stratification [5,7]. Clinically, SSc‑ILD often begins insidiously; exertional dyspnea, dry cough, and bibasal crackles on auscultation are typical early signs, though they may precede functional decline, underscoring the role of proactive screening [8]. Radiologically, early disease is characterized by subtle abnormalities, such as ground-glass opacities and fine reticular changes, predominantly affecting the peripheral and basal lung regions, as shown in Figure 1. As the disease progresses, these initial changes may evolve into extensive architectural distortion and fibrosis, reflecting irreversible lung damage, as illustrated in Figure 2. Given this heterogeneity in disease course, from subclinical lung abnormalities on imaging to progressive fibrotic change, comprehensive longitudinal monitoring is essential to tailor therapeutic interventions and improve survival outcomes [9].

## Review

Pathogenesis

Initial Injury and Triggering Events

The pathogenesis of SSc-ILD begins with damage to the lung parenchyma elicited by multiple environmental insults and intrinsic factors [11]. Environmental triggers have been attributed to occupational exposure, such as silica dust and organic solvents, identifying them as significant risk factors for the development of SSc-ILD. Genetic susceptibility also plays a key role in modulating the biological response to these insults, contributing to a complex and multifactorial disease process [12,13]. The initial injury predominantly impacts pulmonary endothelial and alveolar epithelial cells, triggering a self-perpetuating cascade of microinjuries that progressively compromise normal tissue architecture [14,15]. It is now well established that endothelial injury serves as a hallmark of early stages of the disease, as evidenced by the elevated circulating levels of von Willebrand factor, endothelin-1 (ET-1), and soluble endothelial cell markers. These mechanisms form the biological basis of the biomarkers discussed below, reflecting epithelial injury, immune activation, endothelial dysfunction, and fibrosis [14].

Fibroblast Activation and Myofibroblast Differentiation

It is the integration of multiple pro-fibrotic signals that activate fibroblasts and drive their differentiation into myofibroblasts. Myofibroblasts are the main effector cells that are responsible for excessive extracellular matrix (ECM) deposition. They are distinct from quiescent fibroblasts in that they express α-smooth muscle actin that allows them to confer contractile properties [11,12].

Myofibroblasts in SSc-ILD are derived from multiple cellular sources, including resident lung fibroblasts activated by pro-fibrotic mediators, circulating fibrocytes recruited from the bone marrow, epithelial cells that undergo epithelial-mesenchymal transition, endothelial cells that undergo endothelial-mesenchymal transition, and pericytes surrounding blood vessels. Together, these diverse progenitor populations expand the pool of activated myofibroblasts and sustain the profibrotic remodeling of lung tissue [11,16].

Mechanisms Driving Fibrotic Persistence and Amplification

It is important to note that the pathogenesis of SSc-ILD involves self-dependent and self-amplifying loops that sustain and accentuate the fibrotic response [11,12]. Transforming growth factor-beta (TGF-β) serves as a central mediator in this regard, whereby it promotes fibroblast survival through activation of anti-apoptotic pathways such as PI3K-Akt signaling. This allows for myofibroblast activation to persist and therefore results in continuous ECM production and progressive architectural distortion of lung tissue [11,17].

**Figure 3:**
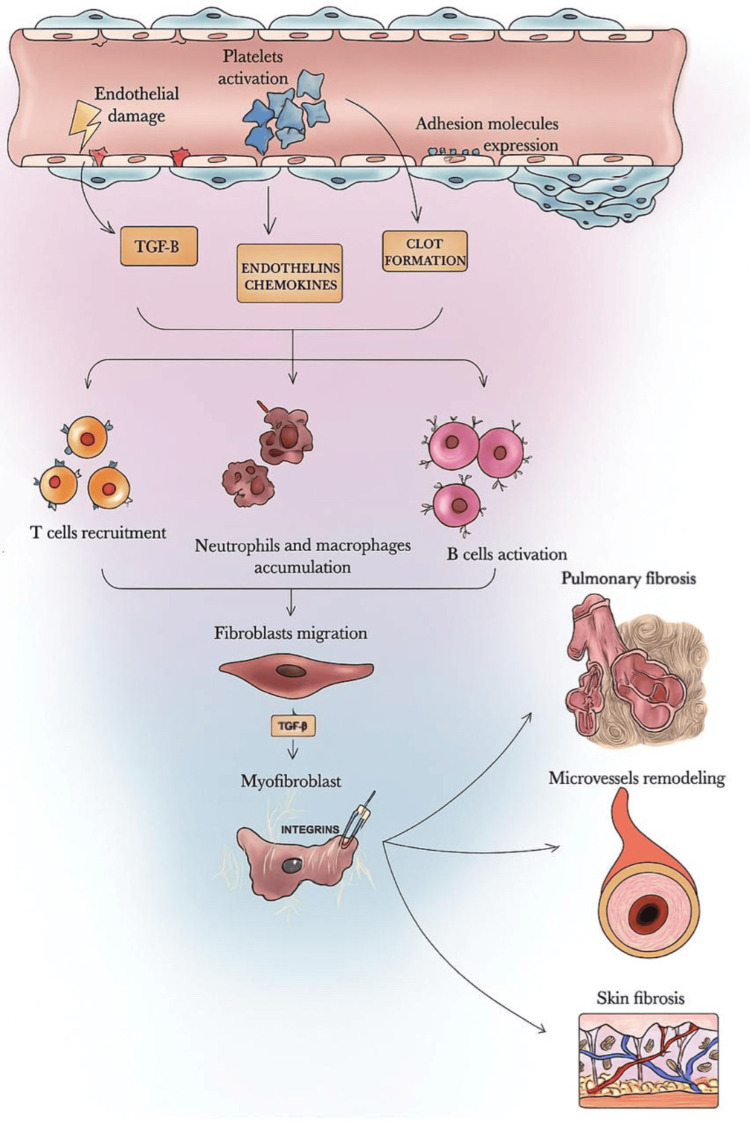
Schematic illustrating the cascade of immune responses that follow initial injury to lung parenchyma, resulting in the eventual fibrotic changes observed in the pathogenesis of systemic sclerosis-associated interstitial lung disease. (1) Repeated injury to alveolar and endothelial cells elicited by environmental triggers, such as oxidative stress, viral infections, silica exposure, and organic solvents, in genetically predisposed individuals. (2) This damage causes the release of pro-fibrotic mediators such as transforming growth factor-beta (TGF-β), endothelins, and chemokines, leading to a robust immune activation. (3) Recruitment of T cells, neutrophils, and macrophages, and activation of B cells follow. (4) Th2 and T follicular helper (Tfh) cells promote M2 macrophage polarization and B-cell activation. B cells produce interleukin-6, TGF-β, and autoantibodies, contributing to the chronic inflammation and fibrosis. (5) Pro-fibrotic macrophages, especially M2 and SPP1-expressing subsets, accumulate and secrete mediators such as TGF-β and platelet-derived growth factor that induce fibroblast proliferation and differentiation into myofibroblasts. (6) Myofibroblasts deposit excessive extracellular matrix, disrupting tissue architecture and promoting fibrosis. Reproduced from Aragona et al. (2022) [19]. Image reproduced from an open-access article distributed under the terms and conditions of the Creative Commons Attribution (CC BY) license.

A 2024 study reported that intercellular communication is also significantly increased in SSc-ILD lungs compared to healthy controls. Key signaling pathways include SPP1-CD44 interactions for macrophage activation, MIF-CD74 signaling between fibroblasts and macrophages, and MDK-mediated autocrine signaling in pro-fibrotic fibroblasts [18]. The overall sequence of injury, immune activation, and fibrotic remodeling in SSc-ILD is summarized schematically in Figure 3.

Systemic sclerosis-associated interstitial lung disease biomarkers

Overview and Clinical Relevance

Biomarkers are indicators of physiological/pathological events or pharmacological responses to therapeutic interventions in an objectively quantifiable manner [20]. In relation to SSc-ILD, biomarkers are critical for differential diagnosis, prognosis, and guiding clinical monitoring and treatment strategies. Biomarkers are classified into the following eight groups: alveolar epithelial proteins; matrix metalloproteinases (MMPs) and tissue inhibitors of MMP; chemokines and cytokines; biomarkers of endothelial activation; circulating cells; connective tissue growth factors; acute-phase proteins; and microRNAs (miRNAs) [7,21]. Each of these groups contributes differently to improving diagnosis, prognostic stratification, and therapeutic monitoring in SSc-ILD.

Alveolar Epithelial Proteins

SSc-ILD is characterized by injury to alveolar epithelial cells, leading to the release of pneumocyte-derived proteins into circulation [7,22]. SP-D levels are often significantly elevated in SSc patients with ILD compared to those without lung involvement or healthy controls [22,23]. However, SP-D alone has not shown a strong or consistent correlation with the extent of lung fibrosis or disease progression in SSc-ILD [7]. SP-A, another surfactant protein associated with alveolar injury, is likewise elevated in ILD but appears to be a less sensitive indicator of SSc-ILD in comparison to SP-D when used for clinical diagnosis or disease monitoring [24].

KL-6 is released by regenerating alveolar type II cells and tends to rise in a range of fibrosing lung diseases due to ongoing epithelial injury [22,24]. Although KL-6 is not specific to SSc, its serum levels are higher in SSc patients with ILD than in those without, and have been explored for diagnostic and prognostic value in clinical practice. Reported KL-6 cutoffs vary widely across cohorts and ethnic populations, limiting universal applicability. In contrast, a recent SSc cohort found that a much higher KL-6 cutoff (~923 U/mL) yielded only ~44% sensitivity (with 85% specificity) for ILD, underscoring the limited sensitivity of KL-6 as a standalone diagnostic test [22,25]. Once ILD is present, KL-6 levels tend to reflect disease severity; they correlate inversely with pulmonary function (e.g., FVC and diffusion capacity) and positively with the extent of fibrosis on high-resolution imaging [25]. Consistently, several studies have linked higher baseline KL-6 to more extensive or progressive lung involvement; for example, elevated KL-6 in early SSc-ILD has been associated with subsequent decline in lung function and with end-stage fibrotic disease [25,26]. Longitudinal observations indicate that dynamic changes in these epithelial markers may track disease activity and treatment responses. Among epithelial biomarkers, KL-6 has the strongest evidence for helping with diagnosis and predicting disease progression, although different studies use different cutoff levels.

Matrix Metalloproteinases and Tissue Inhibitors of Matrix Metalloproteinases

MMPs are proteolytic enzymes responsible for ECM remodeling and are increasingly implicated in the fibrotic processes underlying SSc-ILD. Elevated serum concentrations of MMP-7 and MMP-12 have been consistently observed in patients with SSc-ILD and correlate with greater severity of pulmonary fibrosis, as measured by reduced lung function and radiologic extent [27,28]. Notably, MMP-7 may serve as an early indicator of fibrotic activity, with evidence suggesting its elevation can precede visible interstitial changes on high-resolution imaging and may assist early clinical detection [27]. MMP-7 appears to be a promising early marker of fibrosis, but more studies are needed before it can be used confidently in routine clinical practice.

The regulatory counterpart of MMPs, tissue inhibitors of metalloproteinases (TIMPs), function to maintain homeostatic matrix turnover. Among these, TIMP-1 is the most studied in SSc-ILD. Increased TIMP-1 levels have been reported in affected patients and may reflect underlying alveolar-capillary barrier dysfunction. Some studies have identified a weak but notable inverse correlation between TIMP-1 and DLCO, suggesting its potential role as a biomarker of gas exchange impairment [29]. Although the diagnostic and prognostic strength of TIMP-1 remains limited, its role within the MMP/TIMP axis underscores the importance of protease-antiprotease balance in SSc-related lung fibrosis.

Chemokines and Cytokines

Chemokine ligand 18: The lung’s antigen-presenting cells constitutively produce chemokine ligand 18 (CCL18), and while its exact function is unknown, it appears to be primarily involved in regulating the immune response and cellular trafficking [30]. Fibroblast proliferation and collagen synthesis are also stimulated by CCL18 [30]. During pulmonary fibrosis, native collagen will activate cells in the lungs, such as macrophages and dendritic cells, to produce an increased amount of CCL18 in a positive feedback loop, ultimately causing lung fibrosis [31]. High baseline CCL18 levels independently predict early pulmonary function decline in SSc-ILD, suggesting important prognostic value for clinical risk stratification and disease monitoring [32]. CCL18 has emerged as one of the most consistently supported prognostic chemokine biomarkers in SSc-ILD.

C-X-C motif chemokine ligand 10: C-X-C motif chemokine ligand 10 (CXCL10) is a strong chemokine for Th1 and producers of interferon-gamma (IFN-γ), and increased levels are thought to be related to lung and renal functions [33].

Chemokine (C-C motif) ligand 2: Chemokine (C-C motif) ligand 2 (CCL2) is a chemokine involved in the trafficking of monocytes. During in-vitro experiments with the presence of scleroderma fibroblasts, the suppression of CCL2 prevents the migration of leukocytes [34]. Additionally, CCL2 plays a role in lymphocyte Th2 phenotypic polarization, lymphocyte T trafficking, myofibroblast differentiation, and fibroblast stimulation. Monocytes, type II pneumocytes, and endothelial cells are all primary producers of CCL2 [32]. This chemokine is increased in SSc, and may be a useful predictor of the long-term progression of SSc-ILD [32].

Interleukin-18 and interleukin-6: Early elevated IL-18 and IL-6 are also predictive of subclinical disease progression and may help identify candidates for early treatment [35,36].

Biomarkers of Endothelial Activation

Endothelial damage and activation are a central early event in SSc-ILD. Several circulating markers of endothelial injury and activation have been investigated for their potential use as indicators of early disease or predictors of disease progression [37-39].

Anti-endothelial cell antibodies (AECAs) are circulating autoantibodies targeting endothelial cell proteins, contributing to cellular injury. Serum Immunoglobulin-G (IgG) AECA has been reported to be significantly elevated in 30-54% of patients with SSc when compared to healthy controls [38,39]. AECA positivity in SSc patients was associated with a higher frequency of pulmonary fibrosis, as well as a reduced %FVC and %DLCO [39].

ET-1 is a potent vasoconstrictor peptide [40]. There have been mixed findings regarding its role as a biomarker in SSc-ILD. While ET-1 has been found to be elevated in SSc patients, no significant differences have been observed between those with and without ILD [40]. However, Parker et al. found it to be associated with ILD progression at one year [41]. ET-1 has also been shown to be higher in the bronchoalveolar lavage fluid of patients with SSc compared to those without, but was higher in patients without visible fibrosis on CT than those with [42].

Endothelial-derived extracellular vesicles (EEVs) are derived from the budding of endothelial cell membranes in response to various stimuli, carrying markers of endothelial activation or damage. A study by Colic et al. found that intercellular adhesion molecule 1 (ICAM1)+ extracellular vesicles (EVs) and tissue factor (TF)+ EVs were significantly negatively correlated with %FVC. Additionally, they reported that ICAM1+ EVs were independent predictors of disease progression [43].

Vascular cell adhesion molecule-1 (VCAM-1) is a glycoprotein predominantly expressed on endothelial cells that can be upregulated by pro-inflammatory cytokines such as tumor necrosis factor-alpha [44]. Elevated levels of VCAM-1 have been strongly associated with increased mortality in SSc patients both with and without ILD, except in patients with extensive ILD and pulmonary hypertension at baseline [44]. There is a significant increase in serum soluble vascular cell adhesion molecule (sVCAM) in patients with SSc compared to those without, although there does not seem to be any distinction between patients with and without ILD [44].

Soluble intercellular adhesion molecule-1 (sICAM-1), a cell surface glycoprotein involved in leukocyte adhesion and transendothelial migration during inflammatory and immune responses, has been shown to have higher serum levels in SSc patients when compared to healthy patients. Higher levels are also correlated with ILD and disease severity [45]. Similarly, Pulito-Cueto et al. found increased serum sICAM-1 in SSc-ILD+ patients compared to SSc-ILD- patients [45]. In addition, Colic et al. reviewed the role of ICAM-1-positive EVs, which were found to be elevated in SSc patients with ILD versus those without [43].

Vascular endothelial growth factor (VEGF), a pro-angiogenic growth factor, has been reported to be elevated in the serum of patients with SSc, although it is not specific to SSc-ILD [46]. VEGF expression is also increased in SSc-ILD lung tissue and correlates inversely with pulmonary function, though its specificity is limited [47].

E-selectin, a cell adhesion molecule exclusively expressed by endothelial cells, has been shown to correlate with disease severity in SSc-ILD. Elevated serum E-selectin correlates with reduced lung function and, in some cohorts, with higher mortality [45].

Significantly higher levels of circulating endothelial progenitor cells (EPCs), bone marrow-derived cells that contribute to vascular repair and regeneration of damaged endothelium, have been reported in SSc patients with ILD compared to those without [37]. In SSc-ILD patients, EPC levels were negatively correlated with disease duration. Higher circulating levels have also been associated with more severe ILD [37].

Circulating Cells

Circulating immune cells, particularly monocytes, lymphocytes, and fibrocytes, have been shown to be promising biomarkers for SSc-ILD.

Monocytes

Monocytes are circulating leucocytes that can adopt fibroblast-like characteristics following tissue infiltration [48]. Collagen-producing monocytes of a fibrogenic phenotype, marked by CD14+ and CD45+, are increased in patients with SSc-ILD when compared to healthy controls [48]. Profibrotic CD14+ monocytes found in the serum of patients with SSc-ILD are characterized by the increased expression of activation marker CD163 [48]. Patients with SSc-ILD have an increased monocyte count [49,50]. A study by Bernstein et al. indicated an inverse correlation between SSc-ILD patients’ baseline absolute monocyte count (AMC) and their decline in FVC after 48 weeks. These results suggest that in untreated SSc-ILD patients, AMC may be a useful biomarker of disease progression [49]. However, elevated AMC is not specific to SSc-ILD and has been shown to be a poor prognostic marker for fibrotic disease [50].

Lymphocytes

Lymphocytes are a major class of white blood cells that mediate immune responses and are central to the pathogenesis of SSc‑ILD [11]. In SSc‑ILD, these lymphocyte subsets are significantly dysregulated; an increased Th1/Th2 ratio, elevation in Th17 cells, and a deficiency in regulatory T cells (Tregs) contribute to inflammation, autoimmunity, and excessive collagen deposition [51]. Monitoring subtype distributions and associated cytokines (such as IL‑17, IL‑4, IL‑13, and IFN‑γ) over time could enhance early detection of disease, guide immunomodulatory treatments, and predict therapy response and cytokine studies [51]. Altogether, the presence, phenotype, and activity of lymphocyte subpopulations offer valuable insight into immune imbalance in SSc‑ILD and represent a promising avenue for targeted biomarker development. Given their accessibility in peripheral blood and their dynamic involvement in immune regulation, lymphocyte subtypes represent a functional complement to other circulating biomarkers such as cytokines and acute‑phase proteins [51]. When interpreted alongside chemokine profiles such as CCL2 or IL‑6, lymphocyte phenotyping enhances the multidimensional assessment of immune activation in SSc‑ILD [51]. This integrative approach may support more personalized immunomodulatory interventions and guide treatment timing more precisely.

Connective Tissue Growth Factors

Connective tissue growth factor (CTGF), also known as CCN2, has been found to be increased in SSc patients, with levels correlating with both skin and lung fibrosis. In addition, several CTGF gene polymorphisms have been associated with susceptibility to SSc-ILD [14].

Transforming growth factor-β1 (TGF-β1) is considered a key mediator of SSc pathogenesis. TGF-β1 signalling stimulates fibroblasts and myofibroblasts, which, in turn, secrete TGF-β1 in an autocrine loop [17]. Elevated TGF-β1 levels have also been positively correlated with the degree of pulmonary fibrosis in SSc patients [17].

Platelet-derived growth factor (PDGF) plays a critical role in the fibrotic process in SSc, acting as a potent mitogen and chemoattractant for mesenchymal cells produced by platelets, macrophages, endothelial cells, and fibroblasts. Although PDGF signalling is central to fibrotic mechanisms, PDGF or its autoantibodies have not yet been validated as a practical biomarker in SSc-ILD, and more research is required [14].

MMP-7, a proteolytic enzyme, has been reported to be significantly elevated in the serum and plasma of patients with SSc and is strongly associated with the presence of SSc-ILD [27].

KL-6 is a transmembrane mucin-like glycoprotein secreted by injured type II alveolar epithelial cells [26]. Elevated serum KL-6 levels have been consistently reported in SSc-ILD and are strongly associated with worsening lung disease. Serum KL-6 levels correlate with fibrosis extent and improve with effective therapy [25,26].

SP-D is a pulmonary surfactant lipoprotein secreted by type II pneumocytes that contributes to reducing alveolar surface tension [22]. Elevated serum SP-D levels reflect damage to the capillary-epithelial barrier in ILD and are higher in patients with SSc-ILD compared to those without ILD. Levels correlate with disease severity and fibrosis extent [23,32].

Higher levels of IL-8, a pro-inflammatory cytokine, have been reported in both serum and the bronchoalveolar lavage fluid of patients with SSc-ILD [33]. However, evidence is limited, and further studies are required to clarify its role as a reliable biomarker for SSc-ILD.

Acute-Phase Proteins

Acute-phase proteins are plasma proteins whose concentrations rise (positive acute-phase proteins) or fall (negative acute-phase proteins) in response to systemic inflammation. They are primarily produced by hepatocytes in response to cytokines, most prominently IL-6, and play key roles in modulating host defence and tissue repair processes [33,51].

CRP is the canonical positive acute-phase reactant and is widely used as a non-specific marker of systemic inflammation. In SSc cohorts, higher baseline CRP is associated with more frequent ILD [52]. Multiple observational studies and cohort analyses indicate that elevated CRP correlates with progressive SSc-ILD and poorer outcomes, supporting its role as a pragmatic, readily available prognostic marker. For example, baseline CRP elevation has been linked to long-term ILD progression in early SSc cohorts and to earlier mortality in larger registry analyses [52,53]. However, CRP is not pneumospecific; its levels may rise during systemic flares or multi-organ activity in SSc, including skin involvement, vascular events, or infections, necessitating interpretation alongside lung-specific markers such as KL-6 or SP-D and relevant clinical data to improve specificity for SSc-ILD activity [52]. CRP remains a practical, widely accessible inflammatory marker but lacks specificity for lung involvement. Nevertheless, when combined with lung-specific biomarkers, CRP can contribute to broader clinical assessment and monitoring strategies in SSc-ILD.

IL-6 is an acute-phase cytokine produced by multiple cell types relevant to SSc-ILD, including macrophages, B cells, fibroblasts, and myofibroblasts [33,36]. IL-6 serves as a key upstream driver of hepatocyte acute-phase protein synthesis. Experimental and translational studies demonstrate that IL-6-dependent STAT3 activation drives fibrogenesis across organs, including the lung, and that blockade of IL-6 signaling can modulate these pathways. Clinically, elevated serum IL-6 and IL-6-responsive transcriptional signatures are associated with early functional decline and disease progression in SSc-ILD cohorts [33,36,54], and IL-6-related plasma cell signatures predict progression in independent studies. Although trials evaluating tocilizumab have shown mixed results regarding predictive treatment response [55], IL-6 remains a biologically plausible marker of an inflammatory-fibrotic phenotype that merits careful monitoring [36,54]. Clinical utility of CRP and IL-6 is maximized when they are integrated into a multimodal biomarker panel rather than used in isolation. CRP identifies systemic inflammatory activation and progression risk, while IL-6 provides mechanistic insight and helps stratify patients for targeted therapies [33,36,52]. Longitudinal monitoring is particularly informative: rising CRP over time predicts disease progression, and IL-6 levels or IL-6-responsive signatures can identify patients at higher risk for early decline in FVC, although exact cutoffs vary by assay and cohort [52,54]. Mechanistically, because IL-6 signals through JAK/STAT3 to drive fibroblast activation, its measurement can inform patient stratification for IL-6-targeted or JAK/STAT inhibitory therapies [36,54,56]. The predictive value and specificity of these acute-phase proteins are further enhanced when combined with lung-specific pneumoproteins (KL-6, SP-D), chemokines (CCL18), and cellular biomarkers such as lymphocyte phenotypes and circulating fibrocytes [33]. This multidimensional approach improves risk stratification and guides personalized immunomodulatory or antifibrotic treatment decisions. Recent biomarker frameworks and prediction models for SSc-ILD explicitly endorse this integrated, longitudinal strategy, highlighting the practical utility of combining systemic and lung-specific inflammatory markers to optimize clinical decision-making [33,52,57]. Among acute-phase markers, IL-6 has stronger mechanistic relevance to fibrotic progression than CRP and may help identify patients who could benefit from targeted anti-inflammatory or immunomodulatory therapies [36,58].

MicroRNAs

miRNAs are short non-coding RNAs that post-transcriptionally regulate gene expression and have attracted attention as potential biomarkers in fibrotic diseases because of their stability in biological fluids and involvement in regulating pro- and anti-fibrotic pathways [59,60]. In SSc-ILD, several miRNAs are differentially expressed in blood, lung tissue, and fibroblasts, with some linked to disease severity, progression, and fibrotic mechanisms [59,60].

miR-155 currently has the strongest combined mechanistic and clinical evidence among miRNA candidates in SSc-ILD. It is markedly overexpressed in peripheral blood mononuclear cells and lung tissue of SSc-ILD patients, with higher levels correlating inversely with lung function measures such as FVC and DLCO [60,61]. Experimental data reinforce its relevance; miR-155 mice lacking exposure to bleomycin develop milder fibrosis and have better survival than wild-type controls [60,62]. Functionally, miR-155 promotes fibrosis through macrophage activation and amplification of TGF-β signaling, suggesting both prognostic and therapeutic potential [62].

miR-21 is also consistently elevated in lung tissue and fibroblasts. Induced by TGF-β, it sustains pro-fibrotic signaling by suppressing Smad7, an inhibitory regulator, thereby maintaining ECM gene activation, including COL3A1 and periostin (POSTN). Inhibition of miR-21 in preclinical models attenuates fibrotic remodeling, underscoring its candidacy for therapeutic targeting [57,63].

In contrast, miR-29a is consistently reduced in SSc, particularly within certain autoantibody-positive subgroups. The miR-29 family suppresses transcription of collagen and other ECM components, making it a key anti-fibrotic regulator [60,64]. Decreased circulating miR-29a may contribute to uncontrolled ECM deposition in the lungs. Some evidence suggests that miR-29a measurement could differentiate SSc patients from healthy individuals, especially early in the disease [60,64].

The role of miR-92a is less defined. Its expression varies between studies, influenced by disease subtype and autoantibody profile. While some data indicate reduced serum levels in SSc, associations with ILD presence or severity remain uncertain. miR-92a is implicated in immune regulation and vascular biology, offering indirect relevance to fibrotic pathways [60,65].

miR-4484 is a newer candidate, showing up to 18-fold upregulation in serum from SSc patients versus controls. Preliminary analyses suggest a link to ECM remodeling, but experimental validation is lacking. Additional miRNAs such as miR-138, miR-146b, and miR-101 can downregulate pro-fibrotic gene expression in fibroblasts from SSc-ILD and idiopathic pulmonary fibrosis, highlighting the potential for broader biomarker discovery [60,64,65]. Table 1 presents an overview of key biomarkers in SSc-ILD with an emphasis on their clinical use.

**Table 1:** Overview of key biomarkers in SSc-ILD. Data are summarized from previously published studies. SSc-ILD: systemic sclerosis-associated interstitial lung disease; SP-A: surfactant protein A; SP-D: surfactant protein D; KL-6: Krebs von den Lungen-6; MMP: matrix metalloproteinase; TIMP: tissue inhibitor of metalloproteinase; ECM: extracellular matrix; IL: interleukin; ET-1: endothelin-1; VCAM-1: vascular cell adhesion molecule-1; ICAM-1: intercellular adhesion molecule-1; VEGF: vascular endothelial growth factor; CTGF: connective tissue growth factor; GDF-15: growth differentiation factor-15; FVC: forced vital capacity; CRP: C-reactive protein; miRNA: microRNA; AMC: absolute monocyte count

Biomarker division	Function	Relevance	Examples/Key biomarkers	Potential clinical applications	Associated cells
Alveolar epithelial proteins	Show signs of alveolar cell malfunction or injury. Fast turnover rate and leakage into blood due to capillary-alveolar disruption [7]	Linked to the development and progression of active lung disease [14]. KL-6 is not SSc-ILD specific, but may be helpful in detecting early ILD and in predicting disease progression [25]	SP-A, SP-D, KL-6 [23]	Used for lung illness diagnosis and progression tracking. KL-6 levels ≥400 U/mL are predictive of ILD progression and outperform baseline pulmonary function tests [31]	Type II alveolar epithelial cells [42]
MMP and tissue inhibitors of MMP	TIMPs are involved in the turnover of ECM and control MMP activity to preserve balance [36]	The severity of SSc-ILD and the advancement of fibrosis are correlated with elevated MMPs [37]. Early elevation in MMP-7 may have potential as an early biomarker and precedes radiographic changes [32]	MMP-7, MMP-12, TIMP-1 [33]	May serve as indicators of the degree of fibrosis and the effectiveness of treatment, TIMP-1 levels may correlate with alveolar-capillary function (further validation is needed) [26]	Macrophages, fibroblasts [11]
Chemokines and cytokines	Regulate fibroblast activity, cell movement, and immunological response [12]	Fibroblasts are stimulated by chemokine (C-C motif) ligand 18 (CCL18), while cell migration and fibrosis are impacted by C-X-C motif chemokine ligand 10 (CXCL10) [28] and chemokine (C-C motif) ligand 2 (CCL2) [30], which can exacerbate SSc-ILD	CCL18, CXCL10, CCL2, IL-6, IL-18 [27]	Possible targets for anti-fibrotic and anti-inflammatory treatments [35]. IL-6 and IL-18 may be predictive of early subclinical ILD. CCL2 has a negative correlation with pulmonary function [41]	T cells, macrophages [11]
Biomarkers of endothelial activation	Show how endothelial cells respond to inflammation and fibrosis [43]	Biomarkers of multi-organ fibrosis; the development of fibrosis is influenced by endothelin-1 (ET-1) from activated cells [44]	ET-1	Helpful in determining the risk of fibrosis and endothelial dysfunction [45]	Endothelial cells, vascular smooth muscle cells
CTGFs	Involve cells that move to regions of injury and display features of immune or fibroblast cells [46]	Transforming growth factor-β (TGF-β) and CTGF are important fibrosis mediators that affect lung fibrosis, especially in SSc-ILD	TGF-β, CTGF	Fibrosis severity biomarkers; possible targets for antifibrotic medications [50]. CTGFs may serve as fibrosis severity indicators	Fibroblasts, myofibroblasts
Circulating cells	Essential for fibrosis signaling pathways, promoting the synthesis of ECM and fibroblasts	Monocytes and lymphocytes in lung tissue are indicators of immune system involvement and fibrosis in SSc-ILD [53]	CD14+/CD34+/Collagen type I alpha 1 chain (col1+) progenitor cells [54], Th lymphocytes	May aid in fibrosis detection and disease progression prediction. AMC may help assess FVC decline	Monocytes [11], T lymphocytes [12]
Acute-phase proteins	Proteins that change immunological activity and plasma levels in response to inflammation [6]	Increased levels of SSc-ILD are frequently utilized as markers of systemic inflammation and are associated with worsened lung function [8]	CRP [22]; IL-6 [28]	Prognostic indicators of systemic inflammation [9]. IL-6 may serve as a predictor for FVC decline [41]	Hepatocytes (CRP production); neutrophils (acute-phase activation) [12]
miRNAs	At the RNA level, small non-coding RNAs control the expression of genes	In SSc-ILD, several miRNAs are linked to the course of the disease and may provide targets for treatment	miR-155, miR-29 , miR-92a	New markers for progression and diagnosis; the potential of specific treatments	Various cell types (e.g., fibroblasts, immune cells)

Risk factors

Risk factors of ILD in patients with SSc include the presence of respiratory symptoms, a history of smoking, male gender, diffuse cutaneous systemic sclerosis, being of African American ethnicity, having a longer disease duration, a high erythrocyte sedimentation rate, the presence of anti-topoisomerase I (anti-Scl-70/ ATA) antibodies, an absence of anticentromere antibody, hypothyroidism, and cardiac involvement [66-70]. It is noteworthy that ILD occurs more frequently in diffuse cutaneous SSc, but it can also occur in limited cutaneous SSc [71,72].

Diagnosis

SSc-ILD is often asymptomatic. Common initial symptoms include dry cough, fatigue, and exertional dyspnea [71,72]. In late stages of SSc-ILD, the patient may present with cyanosis and signs of right heart failure. On auscultation, bilateral inspiratory and expiratory “velcro” crackles may be heard in the bases of the lungs [71,72].

There is a spectrum of lung involvement in SSc-ILD, as some patients might have mild disease that is asymptomatic and clinically stable. In contrast, others may have aggressive disease that progresses quickly and can have a significantly worse prognosis. The risk of ILD development in SSc patients is greater in the first four to five years after the initial diagnosis of SSc [71-75].

HRCT is regarded as the gold standard for diagnosing ILD and is very sensitive at detecting ILD. HRCT can detect abnormalities in the lung even when PFTs appear normal. In a Delphi study, there was an agreement that all patients should be screened at baseline using auscultation, HRCT, and PFTs, including FVC and DLCO. Experts agreed on regular, repeated screening utilizing PFTs. When determining the intervals for HRCT screening, the clinician must consider the risk of the patient developing ILD, as there is no consensus on this [76-78].

Non-specific interstitial pneumonia patterns are frequently seen in the HRCT of SSc-ILD with ground-glass opacities and coarse reticulations. However, characteristics of usual interstitial pneumonia may also be seen, including honeycombing and traction bronchiectasis [78-80].

Therapy

Therapeutic regimens for SSc‑ILD now follow a biomarker-informed, personalized medicine approach, combining immunosuppressive, antifibrotic, and biologic treatments based on individual patient profiles and disease progression markers. Mycophenolate [81] remains the preferred first-line therapy, demonstrating efficacy comparable to cyclophosphamide (CYC) with a better safety profile [82,83]. Importantly, recent studies have shown that serial monitoring of pneumoproteins, specifically rises in KL-6 ≥193 U/mL [84,85] or persistently high CCL18 [86], strongly predict progression, and early reductions in KL‑6 or CCL18 after mycophenolate mofetil initiation correlate with improved lung function, underscoring the value of biomarker-guided treatment adjustments [84]. Additionally, composite biomarker panels (e.g., KL-6/SP-D, CA15‑3, ICAM‑1) have shown promise in enhancing risk stratification at baseline, identifying high-risk patients who may benefit from earlier or combination therapies [84,87]. At present, KL-6 and CCL18 are the most clinically actionable biomarkers for monitoring treatment response. Furthermore, predictive biomarker-therapy matching is emerging, with elevated CXCL4 levels signalling immunosuppressive responsiveness [84,86], suggesting a role for CXCL4 in selecting patients most likely to benefit from MMF or CYC [88].

Nintedanib, a tyrosine kinase inhibitor, is a cornerstone antifibrotic: in the trial, it slowed FVC decline by ~44% over 52 weeks, with effects sustained through ~100 weeks [89-91]. Subgroup analysis confirmed efficacy regardless of autoantibody status or MMF background, reinforcing its broad utility. In clinical practice, rising KL‑6 or CCL18, or progressive FVC decline despite immunosuppression, now prompts the early addition of nintedanib, aligning treatment with disease activity instead of preset timelines [89,90].

Tocilizumab (TCZ), an IL‑6 receptor inhibitor, preserves FVC over 48 weeks in early SSc‑ILD [92,93]. Given that IL‑6 levels correlate with fibrotic severity, measuring IL‑6 may help identify the patients most likely to benefit, refining biologic selection [78,93]. Rituximab (RTX), which depletes CD20+ B cells, has shown stabilization or improvement in PFTs, especially in patients with elevated autoantibody levels or B-cell activation, supporting its use when MMF or CYC fail [94,95]. Pirfenidone remains investigational in SSc‑ILD, with inconsistent trial outcomes and no clear FVC benefit [96].

Combination strategies are now guided by integrated biomarker algorithms; MMF remains foundational, with nintedanib added early when biomarkers signal fibrosis, and TCZ or RTX reserved for phenotypes with IL‑6 or B-cell activation. Real-world data suggest concurrent MMF + nintedanib is well tolerated and may outperform monotherapy, particularly in patients exhibiting both inflammatory and fibrotic biomarker signatures [89,90].

Hematopoietic stem cell transplantation) is reserved for rapidly progressive, biomarker-confirmed high-risk disease [97,98]. While exact biomarker thresholds are still under study, candidates often present high IFN‑chemokine scores or sharply rising pneumoproteins [73].

Limitations

SSc-ILD is difficult to detect in its early phases, as it usually presents either asymptomatically or as non-specific symptoms such as exertional dyspnea and a non-productive cough [2,6,14]. While PFTs are non-invasive and widely accessible [78], they can remain normal and may give false negatives [5,9]. PFTs lack sensitivity and are not sufficient for early diagnosis, but may be useful for determining the course of treatment. Currently, the gold standard for diagnosis of SSc-ILD is HRCT, and although HRCT has high sensitivity, it requires exposure to harmful radiation [2,50,84].

Due to the lack of early detection, the diagnosis of the disease is delayed, and patients often face permanent lung damage by the time they begin treatment due to the progression of the disease [6,9]. Patients do not often realize their first symptoms are related to SSc-ILD and do not immediately seek medical care. Universal screening of SSc patients is essential due to the high rate of SSc patients. Additionally, patients often face periods of stable disease, and it is difficult to predict when patients are at risk of disease progression, which occurs in approximately one in three patients and is a predictor of mortality [5,9,58].

There are also inherent issues in finding serum biomarkers. Ideal biomarkers are easily measured and widely applicable [7,35]. As SSc is a disease affecting multiple organ systems, it is difficult to identify a biomarker that is associated singularly with SSc-ILD. Additionally, as the disease itself is relatively rare, finding a patient cohort large enough to provide unbiased results is difficult [7,93].

While the serological pattern of some antinuclear autoantibodies conveys considerable pertinence as biomarkers for detecting the early onset of SSc subsets, outcomes remain relatively futile in the attempt to derive markers that cater to SSc-ILD exclusively [7,91]. Apart from KL-6, a biomarker which has become a clinical reality in Japan, this glycoprotein has been associated with patent increased levels in the presence of lung involvement in SSc [25,65,81]. Although it promises reciprocal findings to ILD progression and stability, polymorphisms in *MUC1*, the gene responsible for KL-6 expression, have been shown to favor a Japanese population in research that examined KL-6 levels among two ethnic cohorts [65,70]. Thus, discrepancies in KL-6 serum cut-off values raise challenges in standardization and call for further research.

Discussion

SSc is a complex and varied condition, with a complex pathophysiology that renders its ILD form especially difficult to track and record for statistical registries. A recent study examining data from 2011 to 2016 in the United States reported overall age- and sex-adjusted incidence and prevalence rates of 1.1 per 100,000 person-years and 7.3 per 100,000 people, respectively. Estimates derived from data across Europe suggest relatively lower disease frequencies, as prevalence estimates were within the range of 1.7 to 4.2 per 100,000 individuals and incidence rates of approximately 0.1 to 0.4 per 100,000 persons. Additionally, ethnic disparities have been continuously recorded over the years, noting an overall noted black African American and Asian preponderance [8,92,93,99].

Many of the biomarkers mentioned are novel for their use in SSc-ILD but have been utilized in other fibrosing lung diseases, such as idiopathic pulmonary fibrosis (IPF), indicating a potential use as a biomarker for SSc-ILD. MMP-7 and CCL18 are both prognostic markers in IPF; serum CCL18 levels have been found to correlate with the severity of fibrosis in IPF and have also been found to be a predictor of ILD progression in SSc [32,71]. SP-D and SP-A in bronchoalveolar lavage fluids have been found to be decreased in patients with IPF, while serum levels are found to be significantly raised and can be used to predict disease activity [23,24].

## Conclusions

SSc-ILD is a rare, complex disease that often causes severe morbidity and mortality. Patients who experience disease progression live with a permanent decline in lung function, drastically decreasing their quality of life and acting as a predictor of mortality. Identifying biomarkers to be used as a diagnostic tool, prognostic indicator, and in the monitoring of the disease is crucial. The use of these biomarkers could allow for early diagnosis and intervention, preserving patients’ lung function and decreasing mortality. More research must be conducted to determine the clinical value of each potential biomarker.
